# Safety and Efficacy of Early Rehabilitation After Stroke Using Mechanical Thrombectomy: A Pilot Randomized Controlled Trial

**DOI:** 10.3389/fneur.2022.698439

**Published:** 2022-04-08

**Authors:** Wei Wang, Ming Wei, Yuanyuan Cheng, Hua Zhao, Hutao Du, Weijia Hou, Yang Yu, Zhizhong Zhu, Lina Qiu, Tao Zhang, Jialing Wu

**Affiliations:** ^1^Clinical College of Neurology, Neurosurgery and Neurorehabilitation, Tianjin Medical University, Tianjin, China; ^2^Tianjin Key Laboratory of Cerebral Vascular and Neurodegenerative Diseases, Department of Neurorehabilitation and Neurology, Tianjin Neurosurgical Institute, Tianjin Huanhu Hospital, Tianjin, China; ^3^Department of Neurosurgery, Tianjin Huanhu Hospital, Tianjin, China; ^4^Department of Rehabilitation, Tianjin Huanhu Hospital, Tianjin, China; ^5^Department of Intensive Care Unit, Tianjin Huanhu Hospital, Tianjin, China

**Keywords:** ischemic stroke, early rehabilitation, mobilization, mechanical thrombectomy, safety, efficacy

## Abstract

**Background:**

Early rehabilitation (ER) has been reported to be both safe and feasible for patients' post-stroke. To date, however, ER-related outcomes concerning patients who have undergone mechanical thrombectomy (MT) have not been investigated. This study aimed to determine the feasibility of ER and whether it improves prognosis in such patients.

**Methods:**

In this single-center, double-blinded, randomized controlled study involving 103 patients who met the study criteria (i.e., has undergone MT), we randomly divided patients (1:1) into ER and conventional rehabilitation groups. The primary outcome was mortality, while secondary outcomes included favorable outcomes (modified Rankin scale of 0–2), the incidence of non-fatal complications, and Barthel Index (BI) scores. We assessed outcomes at 3 months and 1-year post-stroke.

**Results:**

No significant between-group differences were found in terms of mortality and favorable outcomes at 3 months and 1-year post-stroke. At 3 months, 15 (28.8%) patients in the ER group and 29 (56.9%) in the conventional rehabilitation group (*p* = 0.002) had non-fatal complications. The BI in the ER and conventional rehabilitation groups was 100 (85–100) and 87.5 (60–100), respectively, (*p* = 0.007). At 1 year, the incidence of non-fatal complications was similar between both groups [BI in the ER group, 100 (90–100), *p* = 0.235; BI in the conventional rehabilitation group, 90 (63.8–100); *p* = 0.003].

**Conclusion:**

Early rehabilitation (ER) reduces the incidence of early immobility-related complications and effectively improves patients' activities of daily living on a short- and long-term basis. Our results indicate that MT contributes to ER in patients with stroke.

**Clinical Trial Registration:**

www.chictr.org.cn, identifier: ChiCTR1900022665.

## Introduction

Ischemic stroke accounts for 80% of cerebrovascular diseases, and cerebral infarction due to large vessel occlusion has a high fatality and disability rate. In total, 60–80% of patients with ischemic stroke die, have functional dependence despite alteplase treatment, or experience limited treatment efficacy because of low recanalization rates (1–3). In recent years, mechanical thrombectomy (MT) has been actively recommended in randomized controlled trials as the most effective treatment for acute ischemic stroke with large vessel occlusion (4–8). Despite the significant decrease in the mortality of patients undergoing MT, almost 50% present with varying degrees of neurological dysfunction, requiring functional rehabilitation (4, 5).

Rehabilitation is the key treatment to obtain a good functional prognosis in patients with stroke. Early rehabilitation (ER) is recommended in international clinical management guidelines for acute ischemic stroke (6, 7). Despite this, it still remains controversial in practice (8, 9) due to insufficiently conclusive clinical evidence. It has been reported that ER within 24 h of stroke onset did not increase the odds of a favorable outcome, nor did it have a negative effect on mortality rates (10–12). However, it has been reported that ER can reduce the incidence of severe complications, shorten the length of hospital stay for patients with stroke, and improve their ability to perform activities of daily living (13), all of which benefit patients in stroke care units (14).

Early rehabilitation (ER) is unlikely to have an extremely negative effect on stroke outcomes (15). Evidence suggests that ER is feasible for patients admitted to intensive care units and for those with cerebral hemorrhage to improve their functional independence (16–18). At present, there is a lack of research on ER interventions in patients with MT. Consequently, there is a need to assess the safety and efficacy of ER in such patients. We hypothesized that ER within 48 h after stroke onset was feasible and that it would improve functional outcomes for patients with MT at 3-month and 1-year follow-ups.

## Methods and Materials

### Study Design and Patients

A prospective, single-center, randomized controlled study was performed in two groups who were followed up at 3 months and at 1 year with a blind outcome assessment. In total, we enrolled 103 patients with MT who attended our institution from April to September 2019. The study was registered with the Chinese Clinical Trial Registry (Clinical Trial Registration No. ChiCTR1900022665) and was approved by the Ethics Committee of the Tianjin Huanhu Hospital. After patients had been assured of their right to decline participation in our study or withdraw from our study at any time, all participating patients signed an informed consent form.

Inclusion criteria comprised of patients: (i) aged >18 years; (ii) with a history of stroke with an accompanying neurological deficit, confirmed using magnetic resonance imaging (MRI) or computed tomography (CT) scans; (iii) able to undergo MT; (iv) who had a modified Rankin scale (mRS) score <3 prior to the occurrence of stroke; (v) who could understand and execute therapy instructional programs; and (vi) who had no contraindications in terms of commencing rehabilitation within 48 h post-stroke. Exclusion criteria comprised those: (i) unable to undergo cooperative rehabilitation therapy due to severe aphasia, unconsciousness, or cognitive deficits; (ii) with progressive stroke [National Institute of Health Stroke Scale (NIHSS) score that increased ≥4 points 24 h postoperatively]; (iii) with postoperative symptomatic intracranial hemorrhage or massive infarction with midline shift; (iv) with unstable vital signs; (v) with other medical conditions preventing early mobilization, such as severe heart disease, fracture, or other disorders; and (vi) enrolled in another intervention trial or those who declined to provide written informed consent to participate in the study.

Enrolled patients were randomly assigned in a 1:1 manner to an early rehabilitation group (ERG) and a conventional rehabilitation group (CRG) according to a random computer-generated code. Mortality, non-fatal complications, the number of favorable outcomes (mRS, 0–2), and Barthel Index (BI) scores were assessed at 3 months and at 1 year follow-up.

### Intervention

The start time and the plans for rehabilitation intervention differed between the two groups. The CRG group underwent routine rehabilitation treatment only in the stroke unit. Routine rehabilitation was initiated when a patient's condition was relatively stable ≥48 h post-stroke. Routine rehabilitation activities included correct bed positioning, passive and active mobilization in bed, sitting balanced-limb control activities, and activities of daily living. Early out-of-bed mobilization and routine rehabilitation training in the stroke care unit were performed for patients in the ERG. The first out-of-bed activities were started as soon as possible (within 48 h of stroke symptom onset). The following out-of-bed activities were implemented in the ERG: supported or unsupported sitting, transfer with or without assistance, standing, and transfer of feet activities. This group received out-of-bed mobilization therapy for a minimum of 5–10 min per session, with four sessions per day (depending on the patient's tolerance) for ≥4 days a week until discharge. Vital signs were closely monitored during the out-of-bed procedures. Both groups received routine rehabilitation for 30–40 min daily until discharge.

Routine monitoring was continued for the first 3 days. If a patient's vital signs were unstable or if neurological function deteriorated during out-of-bed activities, then the patient was laid flat on the bed and activities were immediately stopped. The program was developed by specialized rehabilitation physicians, while patient instructions and education were provided by specialized rehabilitation therapists and nurses. The patients were unaware of their grouping. The rehabilitation treatment process was supervised by a rehabilitation therapist and a nurse to minimize medical risks.

### Baseline Data

Baseline patient characteristics were collected, including age, sex, risk factors for stroke (hypertension, diabetes mellitus, cardiovascular disease, atrial fibrillation, cerebral infarction, smoking, and alcohol consumption), the NIHSS score, pre-morbid disability, and stroke type. Stroke severity was classified as mild (NIHSS score, <8), moderate (NIHSS score, 8–16), or severe (NIHSS score, >16) (19). The time to first mobilization, total amount of mobilization, and time spent in hospital were also recorded.

### Outcomes

The primary outcome was the mortality rate. Secondary outcomes included the number of patients with a favorable outcome (mRS, 0–2), the incidence of non-fatal complications, and the BI score. mRS usually ranges from 0 to 5, with a score of 6 indicating death. We defined a favorable outcome as an mRS of 0–2 (no/minimal disability) and poor outcomes as an mRS between 3 and 6 (moderate or severe disability, or death). Complications included immobility-related and neurological complications. Immobility-related complications included pneumonia, deep vein thrombosis, urinary tract infection, pulmonary embolism, and neurological progressive and recurrent stroke. Activities of daily living were measured using the BI. BI scores ≥85 were defined as indicating mild dependance or independence (20). Blinded evaluation of both primary and secondary outcomes was undertaken by trained research staff.

### Statistical Analysis

We analyzed data concerning all patients who completed the protocols and the follow-ups. Descriptive statistics were used to analyze all baseline and clinical characteristics. A chi-square test was used to compare differences in categorical variables, a *t*-test was used to compare parametric continuous variables, and a Mann-Whitney *U* test was used to compare non-parametric continuous variables. Logistic regression analysis was used to investigate whether the intervention influenced outcomes at 3 months and at 1 year (adjusted for age, premorbid mRS score, and baseline NIHSS score). All analyses were performed using Statistical Package for Social Science 20.0 (IBM SPSS, Inc., Chicago, IL, USA) software. Statistical significance was set at *p* < 0.05.

## Results

**Figure 1:**
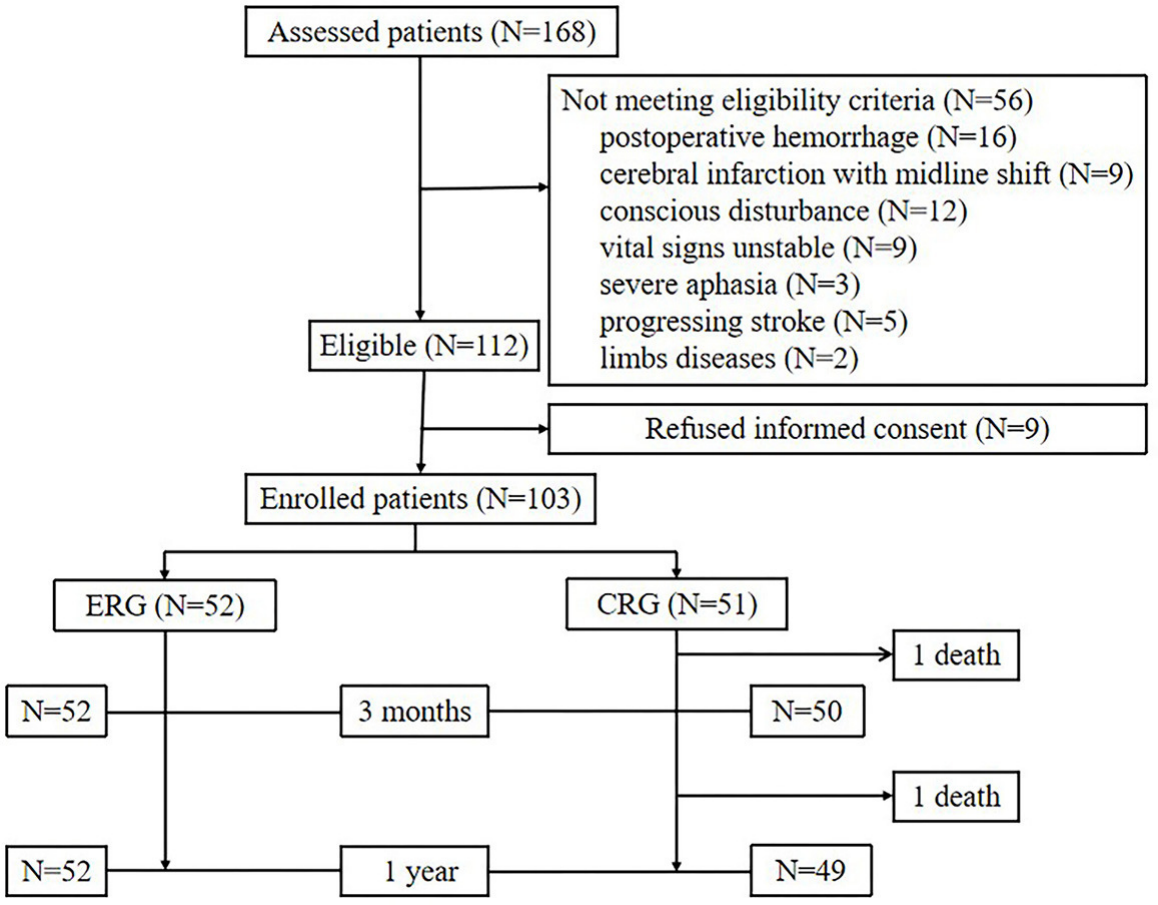
Study profile. ERG, early rehabilitation group; CRG, conventional rehabilitation group.

Between April 2019 and September 2019, 168 patients had undergone MT evaluation, of whom 54 patients did not meet the study inclusion criteria due to postoperative bleeding (*n* = 16), massive infarction with midline shift (*n* = 9), conscious disturbances (*n* = 12), unstable vital signs (*n* = 9), severe aphasia (*n* = 3), progressive stroke (*n* = 5), and limb disease (*n* = 2). Nine patients declined to participate in the study. In total, 103 patients were recruited (Figure 1).

The enrolled patients were randomly assigned into two groups, namely, the ERG and the CRG (*n* = 52 and *n* = 51 patients, respectively). Baseline characteristics between the groups were similar (Table 1). Patients in the ERG commenced mobilization soon after randomization at a median of 42 h (range, 20–48 h) after stroke onset, whereas mobilization in the CRG commenced at a median of 101 h (range, 53–216 h) after stroke onset. This particular between-group difference was significant (*p* < 0.001, Table 2). The time of first mobilization was earlier than 60 h for only two patients in the CRG. The average hospital stay was 11 (range, 7–14) days for the ERG and 15 (9–19) days for the CRG (*p* = 0.002, Table 2). The total duration of mobilization in the ERG was 350 (225–480) min and 240 (150–330) min in the CRG (*p* < 0.001, Table 2).

**Table 1:** Baseline characteristics of enrolled patients.

	**ERG (*n* = 52)**	**CRG (*n* = 51)**	***P-*value**
**Age (years)**	58 (48–66.8)	62 (55–69)	0.077
≤ 65	37 (71.2)	34 (66.7)	0.247
>65	15 (28.8)	17 (33.3)	
**Sex, male**	41 (78.8)	42 (82.4)	0.653
**Stroke risk factors**
Hypertension	29 (55.8)	36 (70.6)	0.119
Diabetes mellitus	9 (17.3)	13 (25.5)	0.311
Cardiovascular disease	9 (17.3)	11 (21.6)	0.626
Atrial fibrillation	6 (11.5)	7 (13.7)	0.738
Previous stroke or TIA	3 (5.8)	7 (13.7)	0.173
Smoking	35 (67.3)	32 (62.7)	0.682
Alcoholics	26 (50)	23 (45.1)	0.695
**NIHSS score**	10 (7.25–12.75)	12 (8–17)	0.123
0–7	13 (25)	11 (21.6)	
8–16	28 (53.8)	26 (51)	
>16	11 (21.2)	14 (27.4)	
**NIHSS score after MT**	8 (5–10.75)	8 (6–12)	0.234
0–7	20 (38.5%)	19 (37.3)	
8–16	28 (53.8%)	27 (52.9%)	
>16	4 (7.7%)	5 (9.8%)	
**rtPA treatment (yes)**	50 (96.2)	50 (98)	0.569
**Pre-morbid mRS**	0 (0–0)	0 (0–0)	0.677
0	49 (94.2)	47 (92.2)	
1	3 (5.8)	4 (7.8)	
2	0 (0)	0 (0)	
**Stroke type**			0.311
anterior circulation infarct	43 (82.7)	38 (74.5)	
posterior circulation infarct	9 (17.3)	13 (25.5)	

*IQR, interquartile range; NIHSS, National Institutes of Health Stroke Scale; rtPA, tissue plasminogen activator; TIA, transient ischemic attack; mRS, modified Rankin Scale*.

*Data are expressed as medians (IQR) and n (%)*.

**Table 2:** Intervention summary and average days of hospitalization in both groups.

	**ERG**	**CRG**	***P-*value**
Time to first mobilization (h)	42 (20–48)	101 (53–216)	<0.001
Total amount of mobilization (min)	350 (225–480)	240 (150–330)	<0.001
Days in hospital (d)	11 (7–14)	15 (9–19)	0.002

*CRG, conventional rehabilitation group; d, days; ERG, early rehabilitation group; h, hours; IQR, interquartile range; min, minutes*.

*Data are expressed as medians (IQR)*.

### Mortality

None of the 52 patients in the ERG died (0%), and only 1 (2%) of 51 patients in the CRG died due to progressive stroke that occurred after 3 months (*p* = 0.997). Similarly, at the 1-year follow-up, there were no deaths in the ERG and only 1 (2%) death among 50 patients in the CRG due to recurrent stroke (*p* = 0.999, Tables 3, 4).

**Table 3:** Outcomes at 3-months.

	**ERG** **(*n* = 52)**	**CRG** **(*n* = 51)**	**OR (95% CI)**	***P-*value**
**Primary outcome**				
Mortality	0 (0)	1 (2)		0.997
**Secondary outcomes**				
Favorable outcome (mRS 0–2)	38 (73.1)	29 (56.9)	1.941 (0.830–4.541)	0.126
**mRS category**				
0	19 (36.5)	11 (21.6)		
1	15 (28.8)	14 (27.5)		
2	4 (7.7)	4 (7.8)		
3	12 (23.1)	10 (19.6)		
4	1 (1.9)	8 (15.7)		
5	1 (1.9)	3 (5.9)		
6	0 (0)	1 (2.0)		
**Non-fatal complications**	15 (28.8)	29 (56.9)	3.740 (1.604–8.718)	0.002
Pulmonary infection	8 (15.4)	18 (35.3)	2.701 (1.020–7.154)	0.046
Vein thrombus	2 (3.8)	8 (15.7)	5.488 (1.112–27.079)	0.037
Urinary infection	1 (1.9)	2 (3.9)	4.270 (0.259–70.421)	0.310
Recurrent stroke	2 (3.8)	5 (9.8)	3.144 (0.542–18.224)	0.201
Progressive stroke	0 (0)	1 (2)		0.997
Vascular occlusion	3 (5.8)	1 (2)	0.247 (0.022–2.827)	0.261
Other adverse events	1 (1.9)	3 (5.9)	1.407 (0.103–19.138)	0.798
**BI**	100 (85–100)	85 (60–100)	0.924 (0.873–0.979)	0.007
≥85	43 (82.6)	27 (52.9)	4.055 (1.595–10.309)	0.003

*BI, Barthel Index; CI, confidence interval; CRG, conventional rehabilitation group; ERG, early rehabilitation group; IQR, interquartile range; mRS, modified Rankin Scale; RR, relative risk*.

*Data are expressed as medians (IQR) and n (%)*.

*All analyses are adjusted for the baseline National Institutes of Health Stroke Scale score, age, and the premorbid mRS score*.

**Table 4:** Outcomes at the 1-year follow-up.

	**ERG (*n* = 52)**	**CRG (*n* = 50)**	**OR (95% CI)**	***P-*value**
**Primary outcome**				
Mortality	0 (0)	1 (2)		0.999
**Secondary outcomes**				
Favorable outcome (mRS 0–2)	43 (82.7)	33 (66)	2.018 (0.771–5.283)	0.153
**mRS category**				
0	26 (50)	12 (24)		
1	10 (19.2)	12 (24)		
2	7 (13.5)	9 (18)		
3	8 (15.4)	10 (20)		
4	1 (1.9)	4 (8)		
5	0 (0)	2 (4)		
6	0 (0)	1 (2)		
Non-fatal complications	3 (5.8)	7 (14)	2.421 (0.563–10.422)	0.235
BI	100 (90–100)	90 (63.8–100)	0.951 (0.920–0.983)	0.003
≥85	47 (90.3)	29 (58)	6.308 (2.104–18.914)	0.001

*BI, Barthel Index; CI, confidence interval; CRG, conventional rehabilitation group; ERG, early rehabilitation group; IQR, interquartile range; mRS, modified Rankin scale; RR, relative risk*.

*Data are expressed as medians (IQR) and n (%)*.

*All analyses are adjusted for the baseline National Institutes of Health Stroke Scale score, age, and the premorbid mRS score*.

### mRS

**Figure 2:**
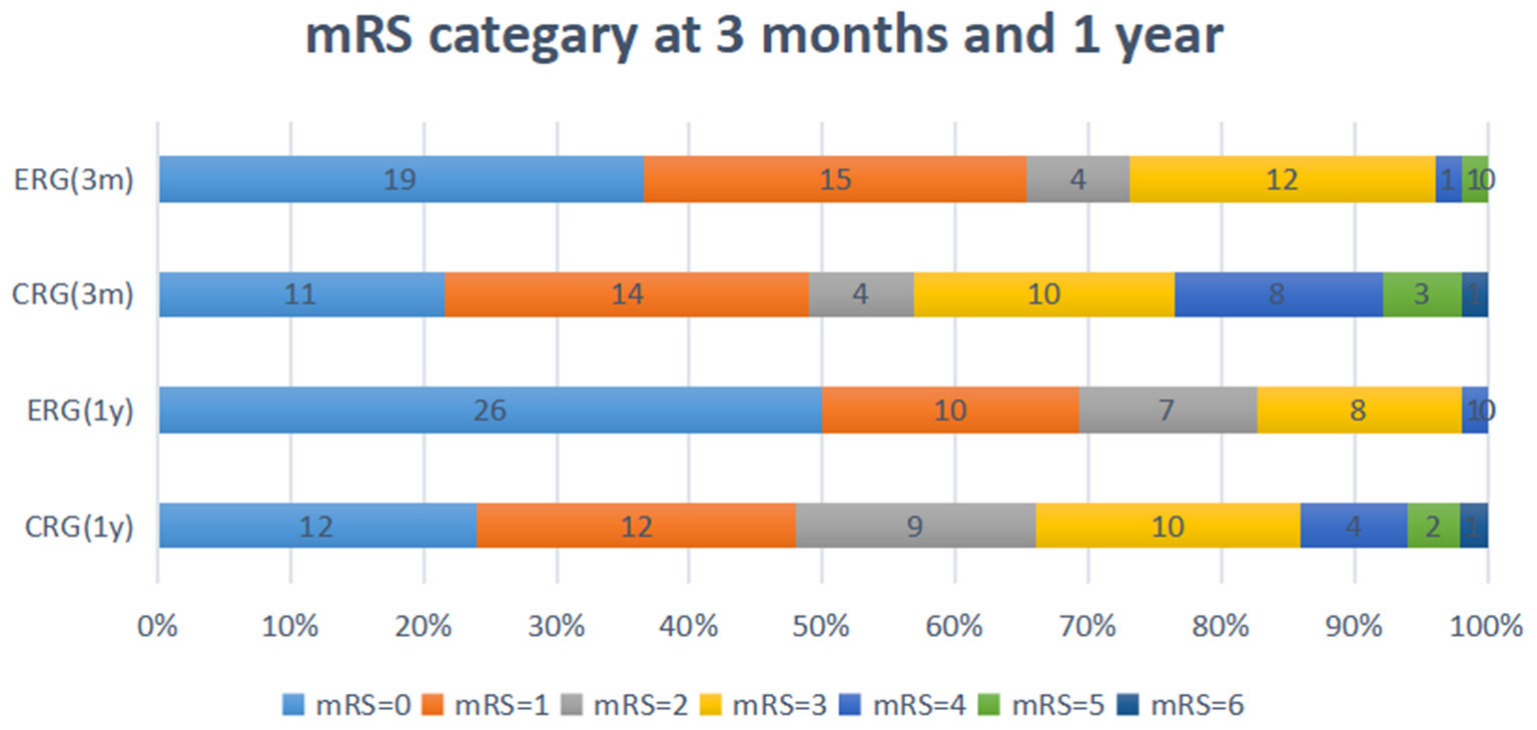
Modified ranking scale (mRS) shift: patients achieving each mRS score at 3-months and 1-year. ERG, early rehabilitation group; CRG, conventional rehabilitation group; mRS, modified Rankin Scale; M, months; Y, year.

Favorable outcomes did not differ significantly between the two groups at the 3-month follow-up [odds ratio (OR) 1.941, 95% confidence interval (CI) 0.830–4.541; *p* = 0.126] or at the 1-year follow-up (OR 2.018, 95% CI.771–5.283; *p* = 0.153). However, the percentage of patients with favorable outcomes (mRS, 0–2) was higher in the ERG at the 3-month follow-up (73.1 vs. 56.9%), and this difference between the groups was maintained at the 1-year follow-up (82.7 vs. 66%, Tables 3, 4). The assumption-free ordinal analysis showed a significant difference between the groups across all mRS scores (0–6, Figure 2).

### Non-fatal Complications

At the 3-month follow-up, there was a significant between-group difference in the number of patients who experienced non-fatal complications in the earlier period of their rehabilitation treatment course. Non-fatal complications occurred in 15 (28.8%) patients in the ERG and in 29 (56.9%) patients in the CRG (OR 3.740, 95% CI 1.604–8.718; *p* = 0.002, Table 3). Immobility-related complications also significantly differed between groups. An in-depth description of the development of immobility-related and neurological complications is shown in Table 3. At the 1-year follow-up, recurrent stroke complications occurred in 3 (5.8%) and 7 (14%) patients in the ERG and CRG, respectively (OR 2.421, 95% CI 0.563–10.422; *p* = 0.235, Table 4).

### Activities of Daily Living

Significant differences in activities of daily living between the two groups were observed at the 3-month and 1-year follow-ups. At the 3-month follow-up (Table 3), the median BI was 100 in the ERG vs. 87.5 in the CRG (OR 0.924, 95% CI 0.873–0.979; *p* = 0.007), and 43 (82.6%) patients in the ERG and 27 (52.9%) patients in the CRG showed mild dependence or independence, respectively (BI ≥85, OR 4.055, 95% CI 1.595–10.309; *p* = 0.003). At the 1-year follow-up (Table 4), the median BI was 100 in the ERG vs. 90 in the CRG (OR 0.951, 95% CI 0.920–0.983; *p* = 0.003), and 47 (90.3%) and 29 (58%) patients in the ERG and CRG showed mild dependence or independence (BI ≥85), respectively (OR 6.308, 95% CI 2.104–18.914; *p* = 0.001).

## Discussion

This was a randomized, double-blind, controlled study on the safety and efficacy of ER in patients who had MT. Our findings showed that postoperative ER did not contribute to increased short- and long-term mortality rates. The incidence of non-fatal complications was significantly lower in the ERG than in the CRG. ER can reduce immobility-related complications, mainly due to a reduction in the incidence of pulmonary infections and venous thrombosis without increasing the incidence of neurological complications. We defined an mRS of 0–2 (minimum or no disability) as a favorable outcome. Although no significant differences between the groups at 3-month and 1-year follow-ups were found, the percentage of favorable outcomes was higher in the ERG than in the CRG. ER improved the patients' abilities to perform daily activities based on our observations at both 3 months and 1 year. Therefore, our study findings provide preliminary clinical evidence of the benefits of ER in patients who had undergone MT.

In preliminary findings, the A very early rehabilitation trail (AVERT) has shown the safety and feasibility of very early rehabilitation for patients with acute ischemic stroke (11, 21). However, this trial lacked clinical evidence concerning ER for patients having undergone MT. In our study, we included patients undergoing endovascular MT in our safety and feasibility assessment and noted the potential benefits of early out-of-bed rehabilitation. Despite showing the feasibility of an early ambulation protocol, no consensus was identified concerning the start time for the intervention. The follow-up AVERT III trial indicated that earlier, more frequent, and more intense out-of-bed activity within 24 h post-stroke was associated with unfavorable outcomes at 3-month follow-up (11). This finding may be related to the timing of ER intervention within 24 h of stroke onset, as some randomized controlled trials regarding early stroke rehabilitation have shown that patients with mild to severe stroke (ischemic and hemorrhagic) may benefit from high-intensity rehabilitation 24 h after stroke occurrence (10, 22). One study from southern Brazil reported that early mobilization had no negative effect on the rates of immobility complications and mortality within 48 h of stroke symptom onset (23). Therefore, based on the above, we set our early out-of-bed activity intervention to within 48 h post-stroke.

Due to cerebral autoregulation impairment, blood pressure changes may aggravate brain tissue reperfusion injury and increase the risk of hemorrhage transformation (24), whereas postural changes may affect the ischemic penumbra area and normal brain tissue blood supply due to residual stenosis of intracranial vessels after surgery, which suggests that an ER rehabilitation intervention for patients undergoing MT may be unsafe (25). However, no fatal complications, such as symptomatic cerebral hemorrhage or progressive stroke, were observed in the ERG. Moreover, there was no significant between-group difference found in terms of mortality. This result is consistent with those of other ER randomized controlled trials (16), which indicates that early out-of-bed sitting is unlikely to have major negative effects on stroke outcomes. Additionally, no similar complications were found to be associated with ER (15, 23). Although ER did not improve the prognosis of patients, it had no serious adverse effects. This result shows that ER is effective for reducing early immobility-related complications in patients who have undergone MT and has guiding significance for the postoperative management of such patients.

The incidence of pulmonary infection and lower limb venous thrombosis was also found to be significantly lower in the ERG than in the CRG. This may be due to the disturbance of consciousness and bed rest after surgery in patients who have undergone MT, which increases the probability of aspiration and hypostatic pneumonia. This, in turn, increases the probability of lower limb venous thrombosis in patients with limb paralysis and immobility (26). Extended immobility has been associated with medical complications during hospitalization, and patients with stroke are more likely to have acute complications which significantly negatively correlates with functional prognoses (27) in the early stages of their hospitalization. ER promotes recovery and reduces immobility-related complications and may, therefore, consequently reduce the length of hospital stay (28, 29). This may explain why patients in the ERG were better able to perform activities of daily living than those in the CRG. Furthermore, early rehabilitation can effectively prevent complications, such as infection, which is a possible reason for low mortality rates found in this study (14). Similar to previous studies (29, 30), our study findings provide further evidence in support of the safety and efficacy of ER (within 48 h postoperatively) commencement after MT. We found that ER can reduce early complications in patients undergoing MT in addition to shortening hospital stay, thereby resulting in a favorable prognosis.

This study had some limitations. First, our study was a single-center trial. Second, as a small pilot trial, it intended to preliminarily explore the safety and feasibility of ER of patients with MT. Critical and unstable conditions are contraindications for early out-of-bed rehabilitation. Therefore, the trial did not include patients with critical and unstable conditions. Although there were some limitations in the study, our trial showed that ER is safe and feasible. However, multicenter randomized controlled trials with larger sample sizes are needed to validate our study findings in future.

In conclusion, ER did not increase the probability of a favorable prognosis for patients undergoing MT. However, ER reduced the incidence of early immobility-related complications, shortened hospital stay, and effectively improved the activities of daily living on a short- and long-term basis without increasing mortality and neurological-related complications.

## Data Availability Statement

The raw data supporting the conclusions of this article will be made available by the authors, without undue reservation.

## Ethics Statement

The studies involving human participants were reviewed and approved by the Ethics Committee of the Tianjin Huanhu Hospital. The patients/participants provided their written informed consent to participate in this study.

## Author Contributions

WW, ZZ, MW, YY, and JW conceptualized and designed the study. WW drafted the initial manuscript and revised the report. JW coordinated and critically revised the study for important intellectual content. YY, YC, HZ, HD, and WH completed the rehabilitation intervention-related work. TZ coordinated with the hospitalized patients. LQ conducted the statistical analysis. All authors contributed to the article and approved the submitted version.

## Funding

This study was funded by the Tianjin Key Medical Discipline (Specialty) Construction Project, Tianjin Key Research and Development Program in Science and Technology (No. 19YFZCSY00260), and Tianjin Health Science and Technology Project (No. MS20015).

## Conflict of Interest

The authors declare that the research was conducted in the absence of any commercial or financial relationships that could be construed as a potential conflict of interest.

## Publisher's Note

All claims expressed in this article are solely those of the authors and do not necessarily represent those of their affiliated organizations, or those of the publisher, the editors and the reviewers. Any product that may be evaluated in this article, or claim that may be made by its manufacturer, is not guaranteed or endorsed by the publisher.

## Supplementary Material

Supplementary Figure 1 Comparison of first mobilization time between two groups. ERG, early rehabilitation group; CRG, conventional rehabilitation group; The abscissa represents the number of patients in the two groups; the ordinate represents first mobilization time of each enrolled patient (hour); The blue and yellow lines represent the fluctuation range of first mobilization time in the early rehabilitation group and the conventional rehabilitation group respectively.

## Abbreviations

BI: Barthel Index
CI: confidence interval
CRG: conventional rehabilitation group
ER: Early rehabilitation
ERG: early rehabilitation group
IQR: interquartile range
NIHSS: National Institutes of Health Stroke Scale
mRS: modified Rankin scale
MT: mechanical thrombectomy
OR: odds ratio
RR: relative risk.
