# Supplementary figures and images for: Safety and Efficacy of Early Rehabilitation After Stroke Using Mechanical Thrombectomy: A Pilot Randomized Controlled Trial
Source: Front Neurol. 2022 Apr 8;13:698439. doi: 10.3389/fneur.2022.698439 (PMC9028453; doi:10.3389/fneur.2022.698439)

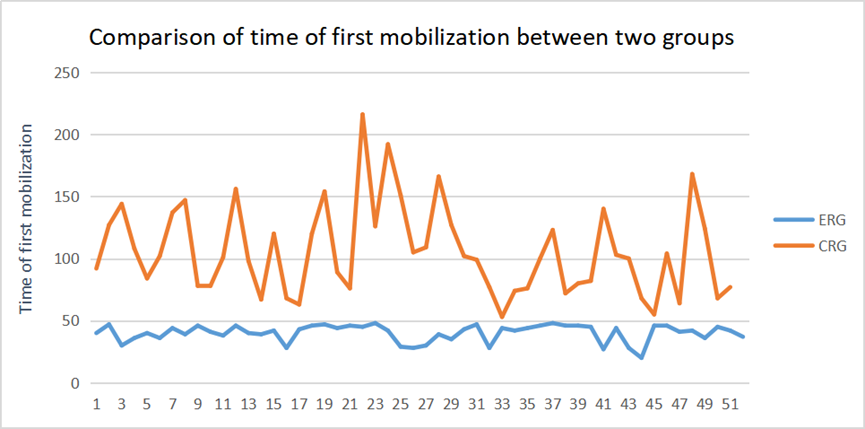

Supplement: Supplementary Figure 1 — Comparison of first mobilization time between two groups. ERG, early rehabilitation group; CRG, conventional rehabilitation group; The abscissa represents the number of patients in the two groups; the ordinate represents first mobilization time of each enrolled patient (hour); The blue and yellow lines represent the fluctuation range of first mobilization time in the early rehabilitation group and the conventional rehabilitation group respectively. [file Image_1.TIF]
